# The status of amur beak gudgeon *Microphysogobio amurensis* (Taranetz 1937) inferred from complete mitochondrial genome analysis

**DOI:** 10.1080/23802359.2016.1266917

**Published:** 2017-01-17

**Authors:** Xi Chen, Jing-hong Li

**Affiliations:** College of Forestry, South China Agricultural University, Guangzhou, China

**Keywords:** Genome, *Microphysogobio amurensis*, *Biwia*, *Rostrogobio*, type species

## Abstract

The complete mitogenome sequence of *Microphysogobio amurensis* has been amplified and sequenced in this study. The overall base composition of *M. amurensis* mitogenome is: A (30%), C (26.8%), G (17.2%), T (26%), with an obvious A + T bias (56%). The assembled mitogenome, consisting of 16605 bp, has 13 protein-coding genes, 22 tRNA genes, 2 rRNA genes, and a control region. To estimate the status of *M. amurensis*, all available mitogenomes of *Microphysogobio* were downloaded from GenBank for phylogenetic analysis. The result strongly supported the traditional taxonomic hypothesis that *M. amurensis* was sister to *M. brevirostris* and *M. koreensis*. However, taxonomic confusion on its generic assignment remains to be further studied, as this study and other relevant molecular studies not only suggested that *Microphysogobio* could be divided in two major clusters (*viz.* the Palearctic species group and the Oriental species group), but also that two allied genera (*Biwia*, *Rostrogobio*) were embedded within the Palearctic species group of *Microphysogobio*.

The genus *Microphysogobio* Mori 1934, generally including a number of diminutive, rheophilic, and benthic cyprinid taxa referred to as dwarf gudgeon, represents as a geographically widespread but poorly diagnosed group and presents many challenges to authors, who hope to address the species level phylogeny of this genus and allies (Tang et al. 2011; Huang et al. 2016). The northerly outrider of the genus, *M. amurensis* (Taranetz 1937) was initially described as a new genus and species *Rostrogobio amurensis* (type location: the middle and lower Amur River, Khanka Lake), but some later authors (e.g. Reshetnikov 2003, Bogutskaya & Naseka 2004) considered *R. amurensis* a junior synonym of *M. tungtingensis* (Nichols 1926), which was described from Tungting Lake, a tributary of Yangtze River, southern China. In contrast to this opinion, Bǎnǎrescu & Nalbant 1966, 1973) gave it a subspecific designation of *M. tungtingensis* and extended its distribution to northeastern China and Russia, excluding the Korean Peninsula inhabited by another two *tungtingensis*-like *Microphysogobio*. Recently, Kottelat (2006) questioned the synonymy of *M. amurensis* under *M. tungtingensis*, and the differentiation was subsequently confirmed by evidence based on the partial region of mitochondrial DNA (Tang et al. 2011). Although these molecular and morphological analyses have revisited the phylogenetics of the genus *Microphysogobio* and advanced our understanding of intrageneric evolution, most are still inconclusively resolved, including the reliable phylogenetic relationships of *M. amurensis*. In this study, therefore, we determined the complete mitogenome of *M. amurensis,* the first representative of the nominal genus *Rostrogobio*, and analyzed its phylogenetic relationships within subfamily Gobioninae.

DNA was extracted from *M. amurensis* (topotypic specimen SCAU 1179906) collected from the Khanka (Xingkai) Lake, Amur River basin (45°11′47″N, 132°14′32″E) and deposited in the collection of South China Agricultural University (SCAU). The complete mitogenome sequence was amplified using long-range PCR and sequenced using the primer-walking strategy.

The complete mitochondrial genome of *M. amurensis* was 16,605 bp in size (GenBank accession number KY228977), which consisted of 13 protein-coding genes (PCGs), 22 tRNA genes, 2 rRNA genes, and a control region. The overall base composition of the entire genome was 30% for A, 26.8% for C, 17.2% for G, and 26% for T and had low G + C content of 44%. All PCGs started with typical ATG codons, except for the *COX1* gene, which started with GTG. Six of the 13 PCGs harboured the truncated termination codon T–– or TA–, three genes stop with TAA (*COX1*, *ATP6*, *ND4L*), four with TAG (*ND1*, *ND2*, *ATP8*, *ND5*). The longest one was *ND5* gene (1836 bp) in all PCGs, whereas the shortest was *ATP8* gene (165 bp). The length of 12S rRNA and 16S rRNA genes were 958 bp and 1689 bp, respectively. The control region was 929 bp in size and located between tRNA-Pro and tRNA-Phe.

To estimate the status of *M. amurensis*, all available mitogenomes of *Microphysogobio* were downloaded from GenBank for phylogenetic analysis. Our molecular phylogeny shows that the putative members of *Microphysogobio* from East Asia appear in two geographically distinct clades with high statistical support, namely the Palearctic clade and the Oriental clade (Figure 1). The Palearctic clade includes the dwarf gudgeons that are widely distributed in Russian Far East, Mongolia, the Korean Peninsula and the nearby mainland of northern China, as well as northern Taiwan Island; while the Oriental clade joins the taxa of southern China (including central and southern Taiwan Island). Strikingly, the species belonging to these two clades have overlapping ranges in the Yangtze River basin and paleo-Min River basin, which comprise the transitional zone between the Palearctic realm and the Oriental realm (Mori 1936; Bǎnǎrescu 1992). The overall genetic architecture further showed that putative members of *Microphysogobio* occurring in northern China and adjacent area (e.g. *M. amurensis*) are more closely related to Japanese endemic *Biwia zezera* (type species of *Biwia* Jordan & Fowler 1903) than to their southern Chinese cousins (e.g. *M. tafangensis*), indicating that both *Microphysogobio* and *Biwia* are taxonomically heterogeneous. This finding is consistent with several phylogenetic results based on mitochondrial and nuclear DNA sequences (Kim & Bang 2010; Tang et al. 2011). Further proof of this are the degree of divergences between each species of *Microphysogobio* ranging from 7% to 15% in the whole mitogenomes of uncorrected pairwise genetic distances, which greatly overlapped the range (9%–14%) observed between *B.* *zezera* and all known sibling species of *Microphysogobio.* As such, the species currently classified under the genus *Microphysogobio* are clearly in a need of taxonomical revision.

**Figure 1.**
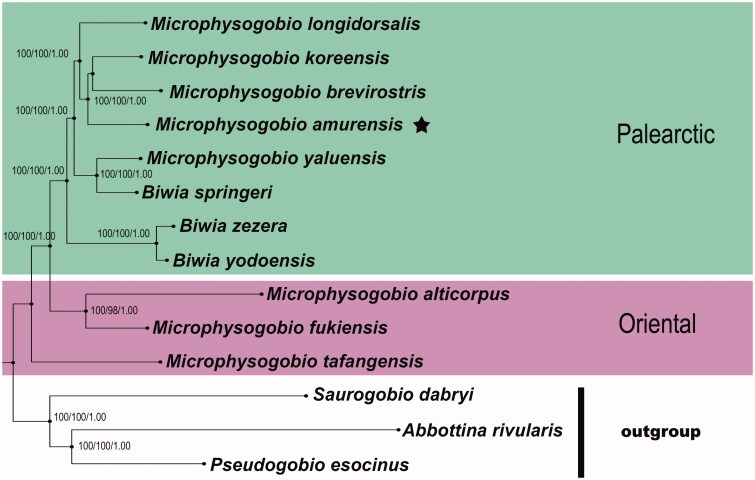
Phylogenetic relationships among *M. amurensis* and other Asian dwarf gudgeons with complete mitogenome sequences on GenBank were inferred using the Maximum-Likelihood (ML), Neighbour-Joining (NJ), and Bayesian (BA) phylogenetic analyses, respectively. Numbers above branches are bootstrap support of the ML, NJ analysis higher than 95 and posterior probabilities of the Bayesian analysis higher than 95%, for the combined analysis. The results for the entire mitogenomes gave with three methods very similar tree topologies. The gene’s accession numbers for tree construction are listed as follows: *M. tafangensis* (KF857260.1), *M. alticorpus* (KC762939.1), *M. fukiensis* (KJ933414.1), *M. brevirostris* (KF319122.1), *M. yaluensis* (KR075133.1), *M. koreensis* (JX179157.1), *M. longidorsalis* (AP011394.1), *Biwia zezera* (AB250107.1), *B. yodoensis* (AB250108.1), *B. springeri* (AP011360.1), *Abbottina rivularis* (AP011257.1), *Saurogobio dabryi* (KF534790.1), *Pseudogobio esocinus* (AP009310.1). Clades with taxa inhabited different biogeographic realms were marked by various shading/colours (Oriental: the Oriental species; Palearctic: the Palearctic species). The internal placements of *Biwia* taxa from Japan and Korea within the genus *Microphysogobio* implied that their independent generic positions were not supported herein, and they probably could be placed in a single genus. If the priority status of *Microphysogobio* vs. *Biwia* is confirmed, the latter being a senior synonym.

Unfortunately, the issue that profoundly influences taxonomic limbo and confounds the systematic of *Microphysogobio* and allied genera to date is loss of the type specimen of *M. hsinglungshanensis* (type species of *Microphysogobio*); even none of the genetic data of *M. hsinglungshanensis* is virtually available for corroborating the validity of *Microphysogobio* as a distinct genus (Jiang et al. 2012; Jiang & Zhang 2013). Recent molecular studies of the Asian gudgeons, however, have examined many of the *Microphysogobio* taxa that have been considered closely related to *M. hsinglungshanensis* (including *M. chinssuensis,* which is regarded as a synonym or counterpart of *M. hsinglungshanensis* by the majority of authors). Their results were in accordance with the aforementioned phylogenetic relationships (Yang et al. 2006; Liu et al. 2010; Tang et al. 2011). According to its original description by Mori (1934), *M. hsinglungshanensis* was known from Hsinglung County, bordering Beijing, northern China that belongs to the Palearctic region in biogeography, hence we could not eliminate the possibility that this name-bearing type species may fall into the Palearctic clade with *B. zezera* and *M. amurensis*. Taken together, it seems probable that the nominal genus *Biwia* consists of the autapomorphic species of *Microphysogobio* (including *Rostrogobio*) and does not warrant separate status.

Here, we choose to recognize ‘*Microphysogobio*’ *amurensis* with this genus in single quotes until the complete taxonomic revision of *Biwia*-*Microphysogobio* complex is elucidated, but we will not be surprised if this species is reallocated into the genus *Biwia* in the future.
